# The Psychometric Properties of the Chinese Version of the Beck Depression Inventory-II With Middle School Teachers

**DOI:** 10.3389/fpsyg.2020.548965

**Published:** 2020-09-29

**Authors:** Xiuna Wang, Yutong Wang, Tao Xin

**Affiliations:** Collaborative Innovation Center of Assessment for Basic Education Quality, Beijing Normal University, Beijing, China

**Keywords:** Beck Depression Inventory-II, reliability, construct validity, measurement invariance, middle school teachers

## Abstract

As previous researchers have found, like other parts of the world, depression is prevalent among middle school teachers in China. The Beck Depression Inventory-II (BDI-II) has been widely used to detect depression among workers in different careers all over the world and has shown good scale properties but inconsistent factor structures. To examine the psychometric properties of the BDI-II among middle school teachers, a nationally representative sample of 4,672 valid cases from 688 middle schools were included. We first generated a new bifactor model based on exploratory factor analysis and agglomerate cluster analysis of the residual item correlations and then validated the modes and examined measurement invariance across gender and school location with multiple-group confirmatory factor analysis (CFA). Results indicated that (1) a new bifactor model with a general factor and two group factors (cognitive–affective group factor and somatic group factor) fitted well to the data [WLSMV χ^2^ = 745.651, df = 173, *P* < 0.001, CFI = 0.983, TLI = 0.979, RMSEA = 0.037; 90% CI (0.035, 0.040)]; Omega values for the three factors varied from 0.88 to 0.92; (2) measurement invariance tests indicated that the BDI-II could equally measure depression of middle school teachers across gender and school location groups. All the findings suggest that the BDI-II is a self-report inventory with good psychometric properties for measuring depression among middle school teachers in China.

## Introduction

Depression is one of the most common mental health problems among teachers of compulsory education (Besse et al., 2015; Tu, 2017; Fu and Zhang, 2019). Previous research shows that teachers’ depression scores were significantly higher than the national norm of Chinese adults in several meta-analysis (Zhang, 2010; Zhao, 2015), and the incidence of different levels of depressive disorders were high, such as 21.2% of the middle school teachers showing significantly depressive symptoms in Fuzhou (Luo, 2017). Similarly high prevalence of different levels of depression among teachers was also found in other countries such as the United States, the United Kingdom, and Mexico (Besse et al., 2015; Kidger et al., 2016; Soria-Saucedo et al., 2018). Prior research indicates that depression can negatively affect individual quality of life, job satisfaction, or well-being, and can even increase the risk of suicide (Ferguson et al., 2012; Tsai, 2012; Bianchi and Schonfeld, 2016). Other studies reveal that depression among teachers can negatively impact their teaching effectiveness as well as students’ mental health and academic performance (Kidger et al., 2016; McLean et al., 2018; Harding et al., 2019; Miller et al., 2019). Literature reviews, meta-analysis, and survey results have documented a slow decline in teachers’ mental health over the past two decades in China (Liu and Liu, 2015; Zhao, 2015; Xiao and Wu, 2018). However, most previous research focused on teachers’ general mental health, with only a few specifically addressing depression. To facilitate greater attention to this topic, it is crucial to have access to a brief, reliable, and valid tool to measure teachers’ depression, which can then enable correct treatment of depression for Chinese middle school teachers.

Among various inventories for depression assessment, the Beck Depression Inventory-II (BDI-II; Beck et al., 1996), has become one of the most widely used instruments to measure depressive symptoms for various populations across different cultures, such as in clinical settings, community samples, and school-based populations, including adolescents and teachers (Boyd et al., 2005; Manian et al., 2013; Wu and Huang, 2014; Desouky and Allam, 2017). Originally developed as the Beck Depression Inventory (BDI) (Beck et al., 1961), the tool was revised with information from the DSM-IV (Boyd et al., 2005) and was designed to assess major components of depressive symptomology (Beck et al., 1996). The scale includes 21 four-point Likert-type items and has been translated into Chinese (Wang et al., 2011; Zhu et al., 2018). Various language versions of the BDI-II have achieved good psychometric properties and have been successfully used with populations from various cultural backgrounds (Sacco et al., 2016). However, previous applications have also documented variable factor structures of the BDI-II with different cultural groups assessed (Manian et al., 2013). Even within the same cultural background, sometimes the factor structure is not identical (Wang et al., 2011; Zhu et al., 2018).

BDI-II included items regarding broad coverage of depression criteria to capture the complex nature of depression; thus, items may measure the common depression and specific depression at the same time, which directly induces difficulties in selecting total scores or subscores as indicator of depression severity (Brouwer et al., 2013). One of popular methods to deal with this issue is to explore the latent factorial structure. For BDI-II, a two-factor structure of depression was originally identified by Beck et al. (1996) consisting of a cognitive factor and a somatic-affective factor, which was the typical factor structure in a psychiatric sample (Manian et al., 2013). Subsequently, a series of factor models—including two- and three-factor solutions as well as hierarchical models—were supported, and the ratio of variance explained by different factors was usually inconsistent across studies (Byrne et al., 2007; Osman et al., 2008; Al-Turkait and Ohaeri, 2010; Manian et al., 2013; Wang and Gorenstein, 2013). Taking the Chinese version of BDI-II for example, several factor structures were found across groups, including (a) a two-factor model of somatic-affective and cognitive factors with depressive patients (Wang et al., 2011); (b) a two-factor model of cognitive–affective and somatic factors with first-year college students (Yang et al., 2014); and (c) a three-factor model of cognitive–affective, somatic, and general depressive symptoms with construction workers (Zhu et al., 2018). Accordingly, items representing factors also differ across studies. Overall, though factor analytic approaches have been applied for the BDI-II in psychiatric and general population groups of various cultures, no study has focused on its application to Chinese teachers of basic education. Furthermore, the disparate results indicate that the BDI-II may have a population-specific factorial structure. For this reason, it is necessary to assess the application of the instrument with such a sample to further understand the factor structure of BDI-II.

Recently, more and more researchers employ bifactor models to examine the structure of the BDI-II and found bifactor models well represented the structure of BDI-II (e.g., Ward, 2006; Al-Turkait and Ohaeri, 2010; Brouwer et al., 2013). Bifactor models consist of a general factor accounting for the majority of the common variance with several group factors with no correlations between factors. Researchers usually constructed bifactor models by simply adding a general factor on N first-order factor models; for example, Brouwer et al. (2013) found that bifactor models performed better than the original correlated first-order factor models. However, the clustering of items in group factors needs further investigations for there are some anomalous cases using this method such as irregular loading patterns (Eid et al., 2017). Cooke and Michie (2001) described procedures for generating bifactor structure based on agglomerate cluster analysis of the index Q3, and there are mounting evidence showing that the method performed well. For example, Patrick et al. (2007) applied it to the bifactor structure generation of the Psychopathy Checklist-Revised using the residual items correlations. Until now, to our best knowledge, there is no application to the test of factor structure on Chinese samples. It is meaningful to apply this method for generating bifactor models of BDI-II on Chinese middle school teachers.

Differences in depression of various population groups such as gender mainly rely on the total raw scores of the BDI-II, which means the measurement accuracy of depression across groups are identical; that is, the BDI-II items are invariant and can measure the same latent construct for various groups. Unfortunately, depression inventories are not often equivalent and symptom clusters vary depending on the population of interest (Reise and Waller, 2009). In fact, if measurement equivalence is not achieved, comparisons of BDI-II scores may not be meaningful because definitiveness is lacking in interpreting the difference attributions (Cheung and Rensvold, 2002; Chen, 2008). Furthermore, other researchers have investigated factorial invariance of the BDI-II by gender, but without consistent results. For instance, factorial invariance was found in South African university students (Makhubela and Debusho, 2016) but not in Chinese-heritage and European-heritage college students (Whisman et al., 2013) or Taiwanese adolescents (Wu and Huang, 2014). To our best knowledge, there are no investigations focusing on differences on latent level. Researchers emphasized the necessity of testing measurement equivalence through multigroup confirmatory factor analysis (CFA) (Byrne et al., 2007; Whisman et al., 2013). Our study evaluates the measurement equivalence of the BDI-II by gender and school location in a Chinese teacher sample and offers implications for future research to fill the research gaps.

The primary purpose of this study was to investigate the psychometric properties of the Chinese version of BDI-II (C-BDI-II) using a nationally representative sample of middle school teachers from Mainland China. At first, we explored the factorial structure that underlies in the scale with subsample 1 and then validated it by comparing the results of CFA with seven competing models provided as proper models in prior research on subsample 2. Additionally, we also evaluated the model fit with alternate statistical indices, including coefficient omega, coefficient omega hierarchical (Omega H), explained common variance (ECV), percentage of uncontaminated correlation (PUC), and construct replicability (H). The second goal of this study was to examine measurement invariance across gender and to test whether there were significant differences of depressive symptoms on latent level across gender.

## Materials and Methods

### Participants

The data for the current study came from a 2014 Chinese national assessment conducted by the National Assessment Centre for Education Quality (NAEQ).^1^ Teachers were selected using a two-stage sampling procedure with unequal probabilities method. In the first stage, using indicators of district level including locations, the ratio of urban to rural students, and information about education and economic development, 140 districts were selected for the whole nation. In the second stage, schools within a particular district were selected according to education quality (good, medium, and poor) and location (city, county, and rural). A total of 668 schools were selected from the districts above. All the head teachers of Grade 8 were asked to answer the questionnaire, the number of whom in each school ranged from 1 to 15 with an average value of 6.82. In all, 4691 teachers participated in the survey, but 19 participants failed to respond to the whole questionnaires and were deleted afterward. This resulted in an effective sample size of 4672. The gender distribution was 45.8% males, 53.5% females, and 0.7% did not report their gender information. The composition of current educational level of the sample was 85.5% bachelor degree, 12.4% college degree or below, and 2.1% master’s degree or above. Moreover, 40.6% of them worked in rural schools while 59.4% worked in urban schools. According to the administration records, teachers were all in good physical condition.

### Measurement

Each participant was asked to respond to the Chinese version of the Beck Depression Inventory-II (C-BDI-II) questionnaire (Wang et al., 2011). The C-BDI-II comprised 21 items rated on a 4-point (0–3) Likert scale, from 0 (“*no symptoms*”) to 3 (“*severe symptoms*, *can barely endure it*”). The summary score, which ranges from 0 to 63 points, reflects overall severity of depressive symptomatology. The higher the summary score, the more serious the depression. Cronbach α coefficient for C-BDI-II first responded by Chinese patients was 0.94. All participants responded to the C-BDI-II according to their life situation during the 2 weeks before the implementation.

### Procedure

All the participants were arranged to respond to the paper-and-pencil self-report questionnaires at the same time in a classroom of their own schools under the supervision of a specially trained educator of local education bureaus. The questionnaire administration took about 30 min. Before the administration, the participants practiced how to respond to the questionnaires at least two times and knew that they were required to fill anonymously and that all the data were just used to provide information for evaluating the overall education quality without feedback to individuals or their schools. The teachers provided assent to participate.

### Statistical Analysis

The data analysis is composed of three parts. First, preliminary analyses were performed using SPSS version 25.0 (IBM Corp, 2017), including outliers screen, descriptive statistical analysis, and the relationships between the items and demographic variables. For the nature of the data with only four ordinal response options, the second part was performed using M*plus* version 7.0 with the robust weighted least squares with mean and variance adjustment (WLSMV) (Muthén and Muthén, 1998–2015). Multiple-group confirmatory factor (MCFA) was used to test the MI (Dolan, 1994; Roger and Jenn, 2004) across gender and school location groups using JASP version 0.12.2 (Wagenmakers et al., 2015) with robust variant of the diagonally weighted least squares (DWLS).

Second, factor analysis was conducted to explore the factor structure of the C-BDI-II: (1) standardized exploratory factor was conducted using a random split half sample (*n* = 2332) to provide information of relationships among items of the C-BDI-II, which was used to evaluate appropriate bifactor models. The criteria to determine the number of factors included the following: minimum average partial method (MAP), parallel analysis (PA), and scree plot (O’connor, 2000; Hayton et al., 2004; Auerswald and Moshagen, 2019). Additionally, the suggestions provided by Hammer and Toland (2016) were taken into consideration that it may indicate that a bifactor structure will best conform when the correlation coefficients between subscales are greater than 0.30 or the ratio of the first eigenvalue to the second eigenvalue in standardized EFA is greater than 3.00. If so, we used the group-average agglomerate cluster analysis of residual matrix of all the C-BDI-II item correlations after removing the first factor to explore an appropriate bifactor structure (Cooke and Michie, 2001; Patrick et al., 2007). Then, goodness of the bifactor model was assessed by CFA. (2) To cross-validate the factor structure of the C-BDI-II, CFA with eight competing models was conducted on the other random split half sample (*n* = 2354). Except the single-factor model (Model A) and the model refining in the current study (Model I), other six multidimensional models originally developed with adult participants and widely used in international research of depression were chosen as competing models. Specifications of these models with the original sample are listed as follows:

*Model A*: the unidimensional model with all 21 items loading on a single factor.

*Model B*: a two-factor model with 12 items loading on somatic–affective factor (Items 4, 10–13, and 15–21), and 9 items loading on cognitive factor (Items 1–3, 5–9, and 14) (Beck et al., 1996; clinical adult outpatients).

*Model C*: a two-factor model with 10 items loading on cognitive factor (Items 1–3, 5–10, and 14) and 11 items loading on somatic factor (Items 4, 11–13, and 15–21) (Huang and Chen, 2015).

*Model D*: a three-factor model with 10 items loading on negative attitude factor (Items 1–3, 5–10, and 14), 6 items loading on performance difficulty factor (Items 4, 11–13, 17, and 19), and 5 items loading on somatic elements factor (Items 15, 16, 18, 20, and 21) (Wu, 2010; college students).

*Model E*: a three-factor model with 10 items loading on negative attitude factor (Items 1–3, 5–10, and 14), 6 items loading on performance difficulty factor (Items 4, 11–13, 17, and 19), and 5 items loading on somatic elements factor (Items 15, 16, 18, 20, and 21) (Zhu et al., 2018; construction workers).

*Model F*: a bifactor model with all the items loading on the general factor and two special group factors: 5 items loading on somatic group factor (Items 15, 16, and 18–20) and 8 items loading on cognitive group factor (Items 2, 3, 5–9, and 14) (Ward, 2006; clinical adult patients and college students).

*Model G*: a bifactor (S.I-1) model with Item 20 (Tiredness or Fatigue) as an indicator of the reference domain to estimate the general factor, 12 items loading on cognitive–affective group factor (Items 1–10, 12, and 14), and 4 items loading on somatic–affective group factor (Items 11, 13, 17, and 19) (Faro and Pereira, 2020; community-dwelling adults).

Following widely accepted practice, model fits for the factor analysis above were assessed by testing multiple fit indices, including chi-square (WLSMV χ^2^), comparative fit index (CFI) and Tucker–Lewis index (TLI), root mean square error of approximation (RMSEA), and its 90% confidence interval (90% CI). Adequate fit was considered if the following criteria were supported: the CFI and TLI were >0.90 and RMSEA was between 0.05 and 0.08; CFI and TLI > 0.95 and RMSEA < 0.05 indicated a good fit model (Hu and Bentler, 1999). Furthermore, because regular chi-square difference tests are not appropriate for non-nested model comparisons, we referred to the practice of Wang et al. (2013) and employed the Bayesian information criterion (BIC) to evaluate these models. The between-model differences in BIC between 6 and 10 show “strong” support that the model with smaller BIC fits better and >10 shows “very strong” support (Raftery, 1995). Since BIC is not given while using the WLSMV estimation method in M*plus*, we use the maximum likelihood (ML) estimator instead (Wang et al., 2013).

Besides the traditional methods for evaluating the structural models like model fits and comparisons with competing models, alternate statistics were used to evaluate the model fit, including coefficient omega, Omega H, ECV, PUC, and *H*. Omega and Omega *H* are useful indices to determine whether the subscales are reliable, how much variance is explained by general/specific factors, and whether it needs to use unit-weighted scores when interpreting the results (Rodriguez et al., 2016b). *H* is brought to assess the likelihood of whether the model can be replicated in future studies (Rodriguez et al., 2016b), and high values of *H* (>0.70) suggests a latent variable is well-defined (Mueller and Hancock, 2001). ECV and PUC in an SEM framework are used in conjunction to evaluate whether it is actually appropriate by using a unidimensional model to multidimensional data (Rodriguez et al., 2016b). Rodriguez et al. (2016a) claimed that when both ECV and PUC are greater than 0.70, the relative bias is little and that it is acceptable to fit multidimensional models in a unidimensional manner.

Finally, measurement invariance tests across gender were conducted with the best-fitting model of the C-BDI-II identified in factor analysis on the total sample. Following Meredith and Teresi (2006), four different levels of invariance—configural (factor structure), metric (factor loadings), scalar (observed variable thresholds), and strict (item error variances)—were analyzed with increasing restrictions. We labeled the model for testing configural invariance as the baseline model and then developed hierarchically nested models for testing equivalence of factor loadings, item observed variable thresholds, and item error variances across gender and school location groups. ΔCFI and ΔRMSEA were used as indices to evaluate invariance test. If the criteria standards (ΔCFI < 0.01 and ΔRMSEA < 0.015) are met, the MI models are accepted (Cheung and Rensvold, 2002; Chen, 2007).

## Results

### Preliminary Analyses

The original sample included 4,691 head teachers, but 19 participants failed to respond to the questionnaires and were deleted afterward. This resulted in an effective sample size of 4,672. Data screening was conducted for outliers, and 0.8% of the participants were identified as having total standardized C-BDI-II scores greater than ±3.00. Because the percentage was considered to be minimal given the large sample size here, outliers were not deleted (Tabachnick and Fidell, 2019). Consistent with previous research with non-clinical samples (e.g., Wu and Huang, 2014), the total scores for the whole sample or subsamples of different gender or school location were non-normally distributed with multivariate normality test using multivariate kurtosis (Mardia’s indexes were between 1386.38 and 1666.24, *P*s < 0.000). As such, WLSMV with M*plus* and robust variant of DWLS with JASP were chosen in the following data analysis. Several items were positively skewed, which was similar to other college student samples or community samples (e.g., Wu and Huang, 2014; Dere et al., 2015; Faro and Pereira, 2020). Descriptive statistics are present in Table 1, including mean, standard deviation, skewness, kurtosis, corrected item-total correlation, and χ^2^/*T*-test of the scores between gender and school location groups.

**TABLE 1:** Descriptive Statistics of C-BDI-II Items (*n* = 4672).

**Items**	**All participants**				**Gender**				**School location**			
					**Male**	**Female**	**χ^2^/*T***	**Cramér’s *V*/Cohen’*d***	**City**	**Urban**	**χ^2^/*T***	**Cramér’s *V*/Cohen’*d***

	***M* (*SD*)**	**SK**	**KU**	***r*_(_*_i_*_–__t)_**	***M* (*SD*)**	***M* (*SD*)**			***M* (*SD*)**	***M* (*SD*)**		
(1) Feeling sad	0.74 (0.78)	0.90	0.41	0.65	0.73 (0.76)	0.75 (0.79)	4.23	0.03	0.72 (0.77)	0.76 (0.79)	4.14	0.03
(2) Pessimism	0.56 (0.85)	1.31	0.56	0.58	0.50 (0.82)	0.63 (0.89)	38.81	0.09	0.53 (0.83)	0.60 (0.88)	8.94*	0.04*
(3) Past failure	0.50 (0.79)	1.43	0.97	0.58	0.44 (0.76)	0.56 (0.83)	26.92*	0.08*	0.47 (0.78)	0.54 (0.82)	9.98*	0.05*
(4) Loss of pleasure	0.59 (0.74)	1.10	0.62	0.64	0.59 (0.75)	0.60 (0.74)	3.21	0.03	0.59 (0.74)	0.60 (0.75)	1.68	0.02
(5) Guilty feelings	0.49 (0.82)	1.30	0.14	0.44	0.47 (0.81)	0.52 (0.84)	3.24*	0.07*	0.48 (0.81)	0.52 (0.84)	4.12	0.03
(6) Punishment feelings	0.37 (0.80)	2.25	4.07	0.56	0.33 (0.76)	0.43 (0.85)	28.70*	0.08*	0.36 (0.80)	0.39 (0.82)	9.20*	0.05*
(7) Self-dislike	0.20 (0.48)	2.77	9.14	0.54	0.17 (0.44)	0.23 (0.51)	15.49***	0.06***	0.19 (0.46)	0.21 (0.50)	3.70	0.03
(8) Self-criticalness	0.45 (0.70)	1.71	3.00	0.48	0.48 (0.73)	0.43 (0.67)	9.78*	0.05*	0.45 (0.70)	0.46 (0.71)	0.44	0.01
(9) Suicidal ideation	0.13 (0.39)	3.74	17.51	0.48	0.12 (0.36)	0.13 (0.41)	4.95	0.03	0.12 (0.36)	0.14 (0.42)	6.26	0.04
(10) Crying	0.44 (1.00)	2.03	2.32	0.51	0.50 (1.04)	0.37 (0.95)	79.30*	0.13*	0.40 (0.96)	0.49 (1.06)	14.05*	0.06*
(11) Agitation	0.67 (0.99)	1.29	0.36	0.49	0.69 (1.01)	0.64 (0.97)	5.34	0.03	0.65 (0.98)	0.69 (1.00)	7.34	0.04
(12) Loss of interest	0.65 (0.89)	1.38	1.12	0.51	0.72 (0.94)	0.57 (0.82)	36.58*	0.09	0.64 (0.88)	0.67 (0.91)	36.58*	0.09
(13) Indecisiveness	0.37 (0.64)	1.68	2.28	0.59	0.35 (0.62)	0.40 (0.67)	8.88*	0.04*	0.35 (0.62)	0.40 (0.67)	3.70	0.03
(14) Feelings of worthlessness	0.40 (0.68)	1.83	3.35	0.58	0.44 (0.69)	0.37 (0.66)	23.00***	0.07***	0.40 (0.66)	0.42 (0.70)	6.67	0.04
(15) Loss of energy	0.27 (0.62)	2.14	3.30	0.58	0.27 (0.62)	0.28 (0.63)	1.53	0.02	0.27 (0.61)	0.28 (0.64)	5.23	0.04
(16) Change in sleeping pattern	0.78 (0.82)	0.90	0.29	0.55	0.78 (0.83)	0.78 (0.81)	2.12	0.02	0.74 (0.80)	0.84 (0.85)	4.12	0.03
(17) Irritability	0.71 (0.70)	0.79	0.63	0.63	0.70 (0.69)	0.71 (0.70)	19.45***	0.07***	0.71 (0.70)	0.73 (0.70)	16.11***	0.06***
(18) Change in appetite	0.54 (0.69)	1.09	0.62	0.60	0.53 (0.68)	0.56 (0.71)	3.60	0.03	0.54 (0.69)	0.55 (0.70)	0.64	0.01
(19) Concentration difficulty	0.14 (0.42)	3.60	14.75	0.31	0.09 (0.35)	0.18 (0.48)	54.99***	0.11***	0.13 (0.41)	0.15 (0.43)	4.26	0.03
(20) Tiredness or fatigue	0.63 (0.73)	1.02	0.70	0.54	0.63 (0.71)	0.64 (0.75)	8.52*	0.04*	0.62 (0.71)	0.65 (0.75)	4.63	0.03
(21) Loss of interest in sex	0.58 (0.81)	1.16	0.27	0.49	0.58 (0.83)	0.58 (0.79)	16.77***	0.06***	0.59 (0.81)	0.56 (0.81)	6.04	0.04
BDI-II total	10.12 (9.20)	1.14	1.25		10.01 (9.04)	10.23 (9.40)	−0.81	−0.02	9.84 (8.99)	10.52 (9.49)	−2.46*	−0.07

BDI-II, Beck Depression Inventory-II; M, mean; SK, skewness; KU, Kurtosis; SD, standard deviation; r(I − t), item-total correlation coefficient; χ2/T, Chi-square test for response options across gender or school location and T-test for the total scores between gender or school location. *p < 0.05, ***p < 0.001.

Considering the influence of demographic variables (gender and school location), the Spearman correlation coefficients between the items and genders in the total sample were calculated, indicating that all the coefficients ranged from −0.105 (Item 7) to 0.147 (Item 21) and most of them were without statistical significance, with a median value of −0.057. The similar trends were found between school location groups (urban: median = −0.049; rural: median = −0.066). In terms of gender, the Spearman correlation coefficients lay in the range of −0.015 (Item 21) to 0.058 (Item 7) with a median value of 0.021. Most of the coefficients of different school location groups were around zero (between −0.015 and 0.058), and the median values for urban and rural schools were −0.005 and 0.030.

To validate the factor structure of C-BDI-II, we randomly split the total sample into two parts (*N*_1_ = 2332, *N*_2_ = 2354) with the random function of SPSS 25.0 (IBM Corp, 2017). There were no significant differences between the two subsamples for gender [χ^2^(1) = 2.12, *P* = 0.15, Cramér’s *V* = 0.02], educational levels [χ^2^(2) = 0.10, *P* = 0.95, Cramér’s *V* = 0.01], and school location [χ^2^(1) = 0.10, *P* = 0.75, Cramér’s *V* = 0.01].

### Factor Structure of the C-BDI-II

**FIGURE 1:**
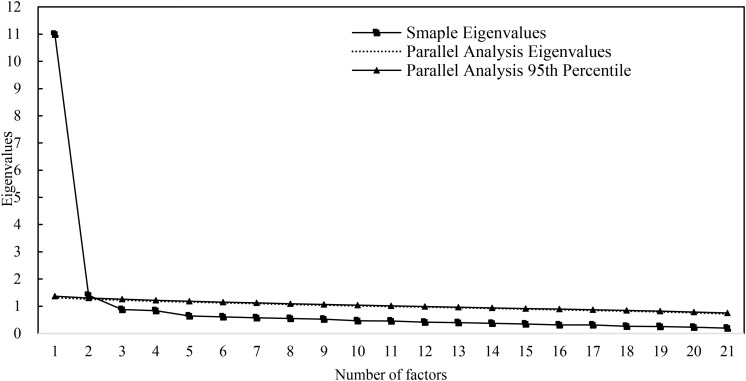
Scree plot for EFA and PA.

To explore the relationships between the items and latent factors, EFA was conducted using a random split half sample (*n* = 2332). As shown in Figure 1, the scree plot shows a predominant first factor and two eigenvalues greater than 1.0. The ratio of the first eigenvalue to the second eigenvalue ranged from 7.83 to 1.02. PA and MAP analysis suggested that extraction of two factors was suitable. The results of the EFA for a two-factor model shows that the model fit achieved adequate level [WLSMV χ^2^ = 2275.300, *df* = 189, *P* < 0.000; CFI = 0.943; TLI = 0.937; RMSEA = 0.069, 90% CI (0.066, 0.071)], all item loadings were greater than 0.40 (*P*s < 0.05), the two factors explained 59.1% of the total variance, and the correlation coefficient between the two factors was 0.71 (*P* < 0.05) (see Table 2 for details). However, considering the results above and the suggestions provided by Hammer and Toland (2016), a bifactor structure may be best performed.

**TABLE 2:** Results of the EFA on Subsample 1 (*n* = 2332).

**Items**	***F1***	***F2***
(1) Feeling sad	0.541*	
(2) Pessimism	0.661*	
(3) Past failure	0.786*	
(4) Loss of pleasure	0.590*	
(5) Guilty feelings	0.697*	
(6) Punishment feelings	0.754*	
(7) Self-dislike	0.897*	
(8) Self-criticalness	0.674*	
(9) Suicidal ideation	0.605*	
(10) Crying	0.568*	
(11) Agitation	0.447*	
(12) Loss of interest	0.466*	
(13) Indecisiveness	0.483*	
(14) Feelings of worthlessness		0.511*
(15) Loss of energy	0.438*	0.426*
(16) Change in sleeping pattern		0.840*
(17) Irritability		0.873*
(18) Change in appetite		0.815*
(19) Concentration difficulty		0.518*
(20) Tiredness or fatigue		0.630*
(21) Loss of interest in sex		0.656*
Eigenvalue (% of total variance)	11.00 (52.40%)	1.40 (6.70%)
Factor correlation	0.705*	

*p < 0.05.

**FIGURE 2:**
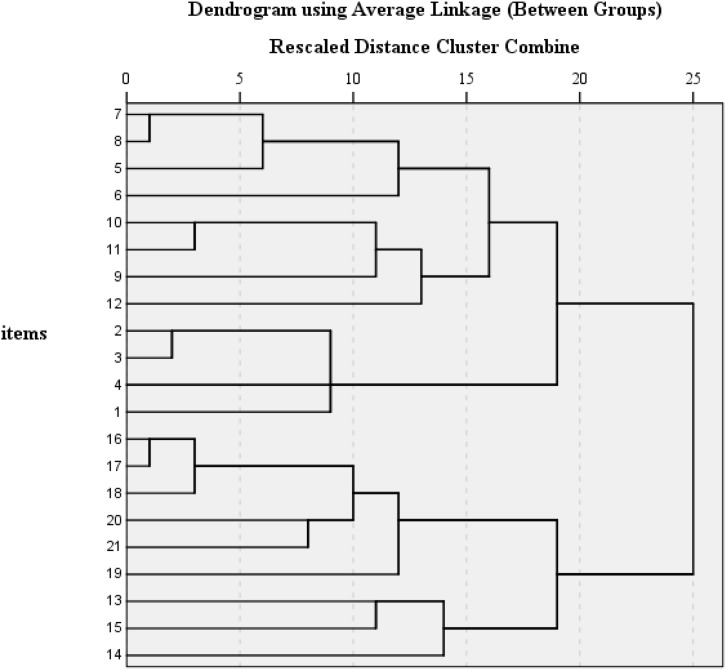
Group-average agglomerate cluster analysis of the residual correlations for C-BDI-II.

The ratio of the first two eigenvalues and the correlation coefficient between the two factors suggested that a common variance underlies all the 21 items of the C-BDI-II, which is the general factor in a bifactor model. To generate the hypotheses of an appropriate bifactor model, we adopted the methods described by Cooke and Michie (2001) and Patrick et al. (2007) to employ the group-average agglomerate cluster analysis of residual matrix of all the C-BDI-II item correlations after removing the first factor. As shown in Figure 2, the result indicated that there were two clear patterns of the residual correlations: the first pattern including the first 12 items referred to the cognitive–affective factor, and the second pattern composed of the remaining 9 items referred to the somatic factor. The patterns here worked as labels indicating relationships between items and group factors (Cooke and Michie, 2001; Patrick et al., 2007). Thus, a bifactor structure was built and then was tested using CFA method.

The CFA result for the bifactor model specified above informed that model fits achieved adequate level [WLSMV χ^2^ = 762.403, *df* = 168, *P* < 0.000; CFI = 0.983; TLI = 0.978; RMSEA = 0.039, 90% CI (0.036, 0.042)], the loadings on the general factor were between 0.432 (Item 19) and 0.779 (Item 15) (*P*s < 0.05), the loadings on cognitive affective group factor ranged from 0.205 (Item 1) to 0.501 (Item 7) (*P*s < 0.05) except four items (Items 9, 10, 11, and 12) with non-significant loadings, and the loading on somatic group factor ranged from 0.098 (Item 13) to 0.484 (Item 18) (*P*s < 0.05) except Item 13 without significant values (see Table 3).

**TABLE 3:** Factor loadings for the bifactor model on two subsamples.

	**Factor loading with subsample 1**			**Factor loading with subsample 2**		
	**(*N* = 2332)**			**(*N* = 2354)**		
**Items**	***G***	***G*_C__–__A_**	***G*_S_**	***G***	***G*_C__–__A_**	***G*_S_**
(1) Feeling sad	0.755***	0.205**		0.751***	0.178***	
(2) Pessimism	0.674***	0.338**		0.709***	0.298***	
(3) Past failure	0.628***	0.481**		0.681***	0.411***	
(4) Loss of pleasure	0.726***	0.231**		0.773***	0.176***	
(5) Guilty feelings	0.497***	0.413**		0.573***	0.344***	
(6) Punishment feelings	0.655***	0.374**		0.722***	0.344***	
(7) Self-dislike	0.623***	0.501**		0.746***	0.395***	
(8) Self-criticalness	0.534***	0.327**		0.596***	0.271***	
(9) Suicidal ideation	0.706***	0.090		0.767***		
(10) Crying	0.724***	0.068		0.733***		
(11) Agitation	0.638***	0.060		0.650***		
(12) Loss of interest	0.666***	0.021		0.704***		
(13) Indecisiveness	0.752***		−0.046	0.789***		
(14) Feelings of worthlessness	0.722***		0.098**	0.719***		0.190***
(15) Loss of energy	0.779***		0.101**	0.803***		0.100***
(16) Change in sleeping pattern	0.610***		0.419***	0.632***		0.484***
(17) Irritability	0.709***		0.409***	0.725***		0.484***
(18) Change in appetite	0.655***		0.484***	0.682***		0.456***
(19) Concentration difficulty	0.432***		0.358***	0.487***		0.299***
(20) Tiredness or fatigue	0.601***		0.400***	0.635***		0.334***
(21) Loss of interest in sex	0.572***		0.286***	0.552***		0.365***

G, general factor; G_C__–__A_, cognitive–affective group factor; G_S_, somatic group factor. **p < 0.01, ***p < 0.001.

To further cross-validate the bifactor structure of the C-BDI-II among Chinese middle school teachers, the same steps of CFA were conducted using the other random split half sample (*n* = 2354). Additionally, seven competing models were taken into consideration. All the results of fit indices of these models using WLSMV estimator are listed in Table 4. As shown in Table 4, all the tested models provided adequate fit indices (CFIs > 0.90, TLIs > 0.90, RMSEAs < 0.08). In general, Model I identified in the current study with a general factor and two group factors and Model G as a bifactor model initially developed by Ward (2006) provided similarly best fit among these alternative models [WLSMV χ^2^ = 745.651, *df* = 173, *P* < 0.001; CFI = 0.983; TLI = 0.979; RMSEA = 0.037, 90% CI (0.035, 0.040); BIC = 91370.907 for Model I; WLSMV χ^2^ = 738.317, *df* = 168, *P* < 0.001; CFI = 0.983; TLI = 0.979; RMSEA = 0.038, 90% CI (0.035, 0.041); BIC = 91407.125 for Model G]. However, the difference of BIC values between Model I and Model G was 36.218 (>10), indicating that Model I performed significantly better than Model G on the data and worked as the best-fitting model. As can be seen in Table 3, factor loadings for the general factor and cognitive–affective group factor and somatic group factor on the second random split half sample were exactly similar to those on the first random split half sample. The ranges of items loading on the three factors were 0.487–0.803, 0.178–0.411, and 0.100–0.484 (*P*s < 0.05).

**TABLE 4:** Goodness-of-fit indices and model comparisons for tested models.

	**WLSMV χ^2^**	***df***	***p***	**CFI**	**TLI**	**RMSEA (90% CI)**	**BIC**
Model A	2071.315	189	<0.001	0.944	0.938	0.065 (0.063, 0.068)	92,348.487
Model B	1804.539	188	<0.001	0.952	0.946	0.060 (0.058, 0.063)	92,144.291
Model C	1809.732	188	<0.001	0.952	0.946	0.061 (0.058, 0.063)	92,123.284
Model D	1670.857	186	<0.001	0.956	0.950	0.058 (0.056, 0.061)	92,016.280
Model E	1051.015	186	<0.001	0.974	0.971	0.044 (0.042, 0.047)	91,596.218
Model F	1414.820	176	<0.001	0.963	0.956	0.055 (0.052, 0.057)	91,782.712
Model G	738.317	168	<0.001	0.983	0.979	0.038 (0.035, 0.041)	91,407.125
Model I	745.651	173	<0.001	0.983	0.979	0.037 (0.035, 0.040)	91,370.907

*WLSMV, weighted least squares with mean and variance adjustment; df, degree of freedom; TLI, Tucker–Lewis index; CFI, comparative fit index; BIC, Bayesian information criterion; RMSEA, root mean square error of approximation; CI, confidence interval; Model A, single-factor model; Model B, the two-factor model in the Beck et al. (1996) study; Model C, the two-factor model in Huang and Chen (2015); Model D, the three-factor model in Wu’s (2010); Model E, the three-factor model in the Zhu et al. (2018); Model F, the bifactor model in Ward’s (2006); Model G, a bifactor (S.I-1) model in the Faro and Pereira’s (2020); Model I, the bifactor model initially identified in the current study.*

Table 5 summarized the results of the five alternative model fit indices including Omega, Omega H, ECV, *H* (an index of construct replicability), and PUC on the two subsamples. Omega values varied between 0.88 and 0.92. Omega *H* for the general factor were 0.93 and 0.94, respectively, and Omega *H* for the group factors ranged from 0.16 to 0.24, indicating that the majority of the reliable variance was attributed to the general factor. *H* values varied with the range of 0.54–0.95 or 0.47–0.95 for the two subsamples. Specifically speaking, the *H* values for the general factor met the criteria (>0.70) provided by Mueller and Hancock (2001), but those for the cognitive–affective group factor or the somatic group factor did not, suggesting that for all the middle school teachers in mainland China, the items of the C-BDI-II give a good definition of the latent depression. Because the *H* values for the cognitive–affective group factor or the somatic group factor were below 0.70, the cognitive–affective factor and the somatic factor do not define the specific depression factor well after excluding the variance explained by the general depression factor. The ECV values for the general factor and PUC values for all the items were all greater than the thresholds of 0.70, informing that it is acceptable to use unidimensional models to fit multidimensional data (Rodriguez et al., 2016a). In all, it provided additional evidence to interpret that there was little bias when fitting the bifactor model to the data of middle school teachers’ responses on the C-BDI-II.

**TABLE 5:** Results of the five alternative model fit indices.

	**ω**	**ω_H_**	***H***	**ECV (%)**	**PUC**
Subsample 1					0.73
*G*	0.88	0.93	0.95	81.32	
*G*_C__–__A_	0.90	0.24	0.58	9.98	
*G*_S_	0.90	0.20	0.54	8.70	
Subsample 2					0.74
*G*	0.90	0.94	0.95	84.51	
*G*_C__–__A_	0.92	0.16	0.47	6.61	
*G*_S_	0.91	0.21	0.57	8.88	

*ω, omega; ω_H_, omega H; ECV, explained common variance; H, construct replicability; PUC, percentage of uncontaminated correlation; G, general factor; G_C–A_, cognitive–affective group factor; G_S_, somatic group factor.*

### Measure Invariance of the C-BDI-II

The bifactor model derived from the factor analysis described above was taken as the optimal model to test the measurement invariance of the C-BDI-II for the whole sample in this study. We first tested whether the construct of depression associated with the same factors and patterns of factor loadings across genders (M0), then tested the equivalence of factor loadings of each item on each factor across groups (M1), and proceeded to test the subgroup observed item threshold differences of each item (M2). Finally, we involved equivalence of item residual uniqueness (M3).

In Table 6, the results of all four models are presented, which examine MI between different genders and school locations. As described below, the models for each level of MI testing had significant DWLS χ^2^, and the other fit indices met the criteria standards (RMSEA ≤ 0.08, CFI ≥ 0.95, and TLI > 0.95), indicating that the models had a high quality of model-data fit. Accordingly, the results for model comparison in pairs informed that the changes of ΔCFI and ΔRMSEA had not achieved the cutoff value of 0.01 and 0.015, respectively. In all, it is inferred that each level of measurement invariance of the C-BDI-II administered to the sample of middle school teachers of different gender and school location groups was supported, and the C-BDI-II items have the same meaning to them.

**TABLE 6:** Model fit indices for measurement invariance testing.

**Model**		**DWLS χ^2^**	***df***	**CFI**	**TLI**	**RMSEA (90% CI)**	**Model comparison**	**ΔCFI**	**ΔRMSEA**
Gender	M0	818.336***	398	0.987	0.987	0.022 (0.020, 0.024)	–	–	–
	M1	838.336***	377	0.989	0.988	0.022 (0.020, 0.025)	M1 vs. M0	−0.001	0.000
	M2	923.305***	398	0.987	0.987	0.024 (0.022, 0.026)	M2 vs. M1	−0.001	0.002
	M3	989.724***	419	0.986	0.986	0.024 (0.022, 0.026)	M2 vs. M1	−0.001	0.000
School location	M0	578.433***	398	0.996	0.996	0.014 (0.011, 0.016)	–	–	–
	M1	549.785***	377	0.996	0.995	0.014 (0.011, 0.017)	M1 vs. M0	−0.000	0.001
	M2	568.433***	398	0.996	0.996	0.014 (0.011, 0.017)	M2 vs. M1	−0.000	0.001
	M3	598.755***	419	0.996	0.996	0.013 (0.011, 0.016)	M3 vs. M2	−0.000	0.000

M0, model for configural invariance; M1, model for metric invariance; M2, model for scalar invariance; M3, model for error variance invariance; DWLS χ^2^, robust variant of diagonally weighted least squares in JASP; df, degree of freedom; CFI, comparative fit index; TLI, Tucker–Lewis index; RMSEA, root mean square error of approximation; CI, confidence interval; ΔCFI, change of comparative fit index relative to the preceding model; ΔRMSEA, change of root mean square error of approximation relative to the preceding model. ***p < 0.001.

Furthermore, differences of the latent factor mean comparisons across gender and school location indicated that comparison with female middle school teachers and male teachers showed lower general depression scores (e.g., *G* score difference = −0.103, *P* = 0.004) and higher cognitive–affective group scores (e.g., *G*_C–A_ score difference = 0.701, *p* < 0.001) and somatic group scores (e.g., *G*_S_ score difference = 0.169, *P* = 0.001) and that there were no significant differences between school location groups (e.g., the latent means for teachers from city schools was fixed to 0 for model identification; *G* score difference = 0.065, *P* = 0.081; *G*_C–A_ score difference = 0.098, *P* = 0.142; *G*_S_ score difference = 0.034, *P* = 0.513).

## Discussion

The purpose of the current study was to evaluate the psychometric properties of the Chinese version of BDI-II in a nationally representative middle school teacher sample from Mainland China. Results suggested that a newly developed bifactor model with two group factors fitted the data best. Additionally, measurement invariance with multigroup CFA was tested and showed that the C-BDI-II had strong measurement invariance across gender.

The newly developed bifactor model was composed of a general factor with factor loadings of all C-BDI-II items and two specific group factors: cognitive–affective group factor with eight items (Items 1–8 on the original scale) and somatic group factor with another eight items (Items 14 to 21 on the original scale). This result is consistent with findings of previous research (e.g., Ward, 2006; Brouwer et al., 2013; Dere et al., 2015; Faro and Pereira, 2020), which suggest that there is a general depression factor accounting for the majority of common variance (e.g., at least 88% in current study) in all items of the BDI-II. It means that it is reasonable to use an overall score when reporting the results with C-BDI-II. The first group factor describes depressive symptoms focused on the cognitive–affective facet, and the second group factor focused on the somatic facet. However, items attached to each of the group factor in the current study are different from the bifactor models from other studies (e.g., Ward, 2006; Brouwer et al., 2013; Dere et al., 2015; Faro and Pereira, 2020). The difference can be interpreted by employing different methods to generate the hypothesis of bifactor models. As known, we followed the suggestions provided by Hammer and Toland (2016) and conducted the group-average agglomerate cluster analysis of residual matrix of all the C-BDI-II item correlations (Cooke and Michie, 2001; Patrick et al., 2007) when exploring the appropriate bifactor structure, but most research referring to bifactor models developed the models by adding a general factor to the N-factor models (e.g., Brouwer et al., 2013). The relationships of item factor in group factors of the current study are similarly consistent with that of Dozois et al. (1998). Furthermore, the cross-validation analysis on the other random split half subsample with seven competing models always supported the conclusion that the bifactor model fitted the data best.

This study also evaluated the bifactor model with alternate statistics. Results provided extra evidence for the goodness of model fit of the bifactor model. The high values of Omega (≥0.88), Omega *H* (≥ 0.93), ECV (≥0.81), *H* (= 0.95) for the general factor, and PUC (≥0.73) all indicated that the general depression factor was well defined and reliably measured, which also suggests that most of the reliable common variance in the observed score attributed to the general depression factor and that it is reasonable to use the total score as an indicator of depression severity. This finding is consistent with the conclusions of prior studies (e.g., Ward, 2006; Brouwer et al., 2013; Dere et al., 2015; Faro and Pereira, 2020). Specifically speaking, 81.32–84.51% of the common variance was accounted for by the general depression factor, and at most, 18.68% was accounted for by group factors; the reliability of the C-BDI-II varied from 0.88 to 0.92. The findings described above are of great importance for practitioners. First, although the general depression factor accounted for the majority of the common variance, it does not mean that it completely invalidates the application of all the group factors; for example, different group factors can be used to design corresponding treatments in the clinical context (Mallinckrodt et al., 2003). Second, practitioners must be careful when interpreting the scores of C-BDI-II because it is hard to differentiate the subscores of group factors from the general construct, and there are high relationships between them.

The second goal of this study was to examine measurement invariance across gender and school location and to test whether there were significant differences of depressive symptoms on latent level across gender. In line with findings of previous research (e.g., Wu, 2010; Wang et al., 2013; Faro and Pereira, 2020), results informed that all models of different levels of invariance across gender groups were satisfied, suggesting that teachers of different subgroups of gender had the same understanding of the latent factors of C-BDI-II and that it is reasonable to directly compare the scores of C-BDI-II among Chinese middle school teachers. Regarding gender differences, it was found that compared to female middle school teachers, males reported a lower general depression score, which is consistent with conclusions in a recent meta-analysis (Salk et al., 2017). For the current study, we also found that male teachers had high cognitive–affective scores and somatic group scores. It is possible that as influenced by traditional Chinese culture, females are more apt to show their symptoms by negative self-evaluation, which may induce them to be depressed (e.g., Hankin and Abramson, 2001; Wu, 2010). There are no significant differences in the three latent factors between middle school teachers of schools located in cities or urban areas.

Notably, although the results of latent factors between teachers of different gender or school location are similar to those with raw total scores, the comparisons of latent factors can provide more information. Taking into consideration the comparison between gender groups, differences of latent cognitive–affective group factor and somatic group factor scores were reported, but not those of general depression factor scores. These findings provided empirical evidence to support that it is more worthwhile to assess differences of the overall scores and specific group factors between groups at the same time especially when previous research gives clues of which particular factor tends to one of the groups (Wu, 2010).

There exist some limitations in the current study. First, all the data used in our analysis were collected within teachers of middle schools, and the findings might not be generalizable to teachers of other educational stages or other professions. Further research needs to be conducted to help validate the findings. Second, we only used one new method to construct bifactor model; other methods such as exploratory bifactor analysis provided by Jennrich and Bentler (2011) should be included to explore the structure of the C-BDI-II. Finally, due to limited resources, we cannot provide criterion-related validity or measurement invariance across with more grouping variables such as age; it is thus necessary to expand related research in the future.

In summary, the results of this study suggest that the Chinese version of BDI-II is a sound self-report inventory with robust psychometric properties for measuring depression among middle school teachers. For the C-BDI-II, the factor structure is well represented by a bifactor model, consisting of a general depression construct and two group factors (cognitive–affective group factor and somatic group factor). Furthermore, male teachers and female teachers shared a common understanding of depression as measured by the C-BDI-II. Overall, this study broadens our knowledge of the psychometric properties of the original C-BDI-II and offers benefits for the broader application of the BDI-II and depression evaluation among general population groups.

## Data Availability Statement

The raw data supporting the conclusions of this article will be made available by the authors, without undue reservation, to any qualified researcher.

## Ethics Statement

The studies involving human participants were reviewed and approved by the Ethics Review Committee of Beijing Normal University. The patients/participants provided their written informed consent to participate in this study.

## Author Contributions

XW and TX designed this study. XW performed the data analysis and interpretation and wrote the first draft of the manuscript. YW contributed to the final manuscript. All authors approved the final manuscript.

## Conflict of Interest

The authors declare that the research was conducted in the absence of any commercial or financial relationships that could be construed as a potential conflict of interest.

## Footnotes

1 The NAEQ, founded in 2007, was an affiliated professional organization of the Ministry of Education of China with the authorization of the State Commission Office for Public Sector Reforms. Its main mission is to develop a comprehensive outlook of educational quality and to conduct national assessments. The NAEQ relies on, and its work is supported by, Beijing Normal University.
